# SARS-CoV-2 Protein S Fusion Peptide Is Capable of Wrapping Negatively-Charged Phospholipids

**DOI:** 10.3390/membranes13030344

**Published:** 2023-03-16

**Authors:** José Villalaín

**Affiliations:** Institute of Research, Development, and Innovation in Healthcare Biotechnology (IDiBE), Universitas “Miguel Hernández”, E-03202 Elche, Spain; jvillalain@umh.es; Tel.: +34-648-891-404

**Keywords:** fusion peptide, membrane fusion, molecular dynamics, phospholipid binding, SARS-CoV-2

## Abstract

COVID-19, caused by SARS-CoV-2, which is a positive-sense, single-stranded RNA enveloped virus, emerged in late 2019 and was declared a worldwide pandemic in early 2020 causing more than 600 million infections so far and more than 6 million deaths in the world. Although new vaccines have been implemented, the pandemic continues to impact world health dramatically. Membrane fusion, critical for the viral entry into the host cell, is one of the main targets for the development of novel antiviral therapies to combat COVID-19. The S2 subunit of the viral S protein, a class I membrane fusion protein, contains the fusion domain which is directly implicated in the fusion mechanism. The knowledge of the membrane fusion mechanism at the molecular level will undoubtedly result in the development of effective antiviral strategies. We have used all-atom molecular dynamics to analyse the binding of the SARS-CoV-2 fusion peptide to specific phospholipids in model membranes composed of only one phospholipid plus cholesterol in the presence of either Na^+^ or Ca^2+^. Our results show that the fusion peptide is capable of binding to the membrane, that its secondary structure does not change significantly upon binding, that it tends to preferentially bind electronegatively charged phospholipids, and that it does not bind cholesterol at all. Understanding the intricacies of the membrane fusion mechanism and the molecular interactions involved will lead us to the development of antiviral molecules that will allow a more efficient battle against these viruses.

## 1. Introduction

An outbreak of the life-threatening respiratory infection, COVID-19, caused by the severe acute respiratory syndrome β-coronavirus 2, SARS-CoV-2, a single stranded positive sense RNA enveloped virus, emerged at the end of 2019 and was declared a worldwide pandemic at the beginning of 2020 [1]. From the beginning of that year, there have been more than 600 million cases and more than six million deaths in the world (https://coronavirus.jhu.edu/map.html (accessed on 15 December 2022), Coronavirus Resource Centre, John Hopkins University). Although new vaccines have been implemented, the pandemic goes on through the rapid evolution of different variants, and it dramatically impacts all aspects of everyone’s life. Although there are several coronaviruses (CoVs) which are relatively mild, there are, apart from SARS-CoV-2, other highly contagious β-coronaviruses which in the past have also produced pandemic outbreaks, including SARS-CoV-1 and Middle East respiratory syndrome coronavirus [2]. SARS-CoV-2 cell entry can occur in two different ways, fusion with either the plasma membrane or with the endosomal one [2,3,4,5]. Membrane fusion, essential for viral entry into the host cell, has been and is one of the main targets for the development of novel antiviral therapies to combat COVID-19.

**Figure 1:**
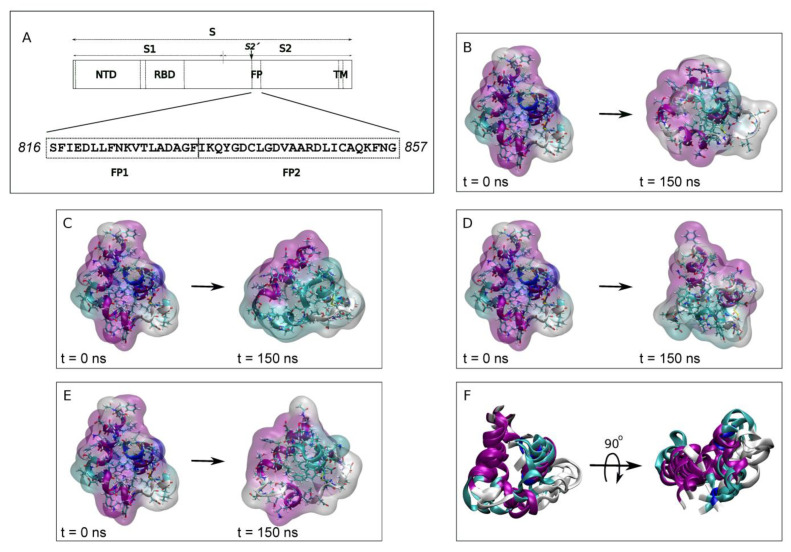
(**A**) Subunits, domains and fusion peptide sequence of SARS-CoV-2 spike protein. NTD, N-terminal domain, RBD, receptor binding domain, FP, fusion peptide and TM, transmembrane domain. (**B**–**E**) Initial (left, t = 0 ns) and final (right, t = 150 ns) peptide structures of FP in water for (**B**,**C**) system 1-1 and (**D**,**E**) system 1-2 (two peptides per system). The peptides are shown in both ribbon and surface transparent drawing style with coloured secondary structures (white (coil), violet (helix), yellow (extended), light blue (turns)). The water molecules and the chloride, sodium and calcium ions have been removed for clarity. (**F**) Structural alignment of the four final peptide ribbon structures.

The main structural components of CoVs include the spike (S), membrane (M) and envelope (E) proteins, the nucleoprotein complexed with the RNA, and the membrane envelope. Similar to other enveloped viruses, the S protein, classified as a class I membrane fusion protein, has been directly implicated in the infectivity of SARS-CoV-2, firstly by binding to the receptor and secondly by entering into the host cell by membrane fusion, the key step in viral infection [2,4,5,6,7]. The coronaviral S protein is embedded in the viral membrane through its transmembrane domain, the N- and C-terminal domains exposed to the extra and intraviral spaces. Spike protein S, a heavily glycosylated protein, forms a homotrimer on the viral membrane and protrudes from it to form the distinctive crown-shaped appearance [8]. The large extraviral domain of the S protein comprises the S1 and S2 subunits: the N-terminal S1 subunit contains the receptor binding domain, while the S2 subunit contains the fusion domain (fusion peptide, FP) which has an essential role in the membrane fusion between the viral and host membranes [4,7,9,10] (Figure 1A). Compared to the rapidly evolving S1 subunit, the S2 subunit, where the FP resides, is highly conserved [11,12]. The heptad repeat domains 1 and 2 of S2 associate to form a six-helix bundle fusion core, and proteolysis at the S2′ site frees the FP (Figure 1A), the region immediately downstream of the S2′ cleavage site, which initiates membrane fusion [4,5,6,9,10,13,14]. Conformational changes in the S protein expose the FP, which interacts with the host lipid bilayer; these changes leading to the membrane fusion have been thoroughly studied but the mechanism is not yet understood.

The exact FP sequence of coronaviruses has not yet been conclusively identified, although it is supposed to reside at or near to the N-terminus of the S2′ protein subunit where the expected characteristics for a fusion domain are found [3,12,15,16,17,18]. Various sequences have been suggested that exhibit conserved motifs, interact with specific membrane lipids, induce membrane leakage or modulate the membrane biophysical properties. The general consensus is that proteolysis at residue ^816^Ser of SARS-CoV-2 forms the mature N-terminus [14,19]. Two relatively well conserved sequences can be distinguished downstream this cleavage site: the FP1 (^816^SFIEDLLFNKVTLADAGF^833^) and the FP2 (^834^IKQYGDCLGDVAARDLICAQKFNG^857^) [12,15,16,20] (Figure 1A). Significantly, FP1 retains the LLF motif and FP2 contains a pair of highly conserved cysteines which seem to be essential for membrane fusion [12,15,16,19,20,21]. It has been suggested that the disulphide bond stabilizes the FP helical structure, increases its binding to the membrane and enables the membrane binding of its two FP1 and FP2 segments in an independent way [3,22,23]. In the same way, FP hydrophobicity determines its ability to interact with the membrane [24]. The SARS-CoV-2 FP modulates the membrane dynamics, decreasing the fluidity of phospholipids, a phenomenon seeming to be modulated by the presence of Ca^2+^ [19,20,25,26]. Interestingly, the FP from SARS-CoV-2 possesses two CHOL-recognition motifs, suggesting that it could bind CHOL at some point in the fusion process [27]. Both FP1 and FP2 seem to be necessary for fusion to take place [17,25]. Significantly, both FP1 and FP2 seem to bind the membrane independently of each other and might act in a cooperative form, promoting each other’s membrane binding [3,22,23,25]. The preferential binding of each one of the peptide sequences might depend on lipid composition and/or lipid specificity, but eventually all FP sequences, which includes the FP1 and FP2 sequences forming a bipartite membrane interaction platform, would bind and insert into the membrane [3,20,28]. 

The structure of FP, which is relatively hydrophobic and intrinsically disordered, has not been determined by x-ray crystallography; however, its structure has recently been solved in bicelles by NMR (RSCB code 7MY8 [19]). According to this work, the FP inserts into the membrane as a wedge [19]. Knowledge of the mechanism of membrane fusion at the molecular level and the specific interactions which take place in the membrane will result in the development of effective antiviral strategies which will make possible inhibition of the virus-cell interaction [11]. It is important to note that there is no information about the structure and/or dynamic properties of the FP role in membrane insertion, interaction and modulation leading to the ultimate viral-cell membrane fusion. Furthermore, membrane fusion is not a spontaneous process since several energy barriers must be overcome before membrane fusion occurs [29,30]. The current SARS-CoV-2 pandemic has highlighted the need to search for broad antiviral biomolecules against this virus and other viruses, and membrane fusion is one of the most significant targets to search for inhibitors that target membrane fusion. 

We have used all-atom molecular dynamics (MD) to study the interaction of the SARS-CoV-2 fusion peptide with specific phospholipids in two different systems: a membrane composed of only one phospholipid plus cholesterol and a model membrane like that of the late endosomal one. The phospholipids that we have used in this work are the following ones: 1-palmitoyl-2-oleoyl-sn-glycero-3-phosphatidylcholine (POPC), 1-palmitoyl-2-oleoyl-sn-glycero-3-phosphatidylethanolamine (POPE), 1-palmitoyl-2-oleoyl-sn-glycero-3-phosphoserine (POPS), 1-palmitoyl-2-oleoyl-sn-glycero-3-phosphoinositol-3-phosphate (PI-3P), N-stearoyl-D-erythro-sphingosylphosphorylcholine (PSM) and sn-(3-oleoyl-2-hydroxy)-glycerol-1-phospho-sn-1’-(3’-oleoyl-2’-hydroxy)-glycerol (BMGP, bis(monoacylglycero)phosphate). It should be recalled that the late endosome is characterized by a low pH and its membrane is characterized by the relatively high content of the negatively-charged phospholipid BMGP [31]. BMGP, which is not found either in the early endosomal membrane nor in the plasma membrane, represents about 15% of its total lipid content and together with phosphatidylinositol, appears to be required for membrane fusion [32,33,34,35,36]. Using a membrane containing only one of these phospholipids plus cholesterol (CHOL) and a model membrane comprising all of them, we have examined the binding and interaction propensity of the FP. Our results show that FP is capable of binding to the membrane, its secondary structure does not change significantly upon binding, it tends to bind preferentially electronegatively charged phospholipids, it is capable of wrapping them, and, interestingly, it does not bind CHOL. Understanding the intricacies of the membrane fusion mechanism as well as the molecular interactions between proteins and lipids will surely lead us to the development of antiviral molecules that will allow an effective battle against these viruses.

## 2. Experimental Section

Unrestrained all-atom MD simulations were conducted using NAMD 2.14 [37] with the CHARMM36 protein and lipid force fields [38,39,40]. All MD parameters used in this work have been described previously [41,42]. The temperature was 310 K. The whole systems, comprising water, ions, membrane and peptides, were equilibrated before each one of the MD simulations for 5 ns after 100,000 steps of minimization so as to remove unfavourable atomic contacts. In the equilibration step, the peptides were constrained, but all the other molecules, including membrane lipids, water and ions, were completely allowed to move so that they could fit completely in the system. The production trajectory for step one (system 1) was computed for 150 ns, for step two (system 2) for 450 ns and for step 3 (system 3) for 800 ns (see below).

The fusion peptide (FP) structure pertained to PDB code 7MY8 [19]. The fifteen structures submitted [19] were averaged and afterwards, the final structure was minimized. All membrane systems were built using the Charmm-Gui web server (http://www.charmm-gui.org, accessed on 2 December 2022 [43]). Afterwards, the peptides were placed and combined with the previously created specific membranes, the peptide/membrane systems were solvated, water inside the bilayer was removed and, finally, ions were added. Each of the complete systems 1 and 2 contained two FP peptides, a membrane consisting of two monolayers, water and ions to an equivalent concentration of 0.15 M in a rectangular box (neutral environment). Its height and cross-sectional area were allowed to vary independently of each other. All the systems contained an excess of water [44]. The lipids we have used in this work are 1-palmitoyl-2-oleoyl-sn-glycero-3-phosphatidylcholine (POPC), 1-palmitoyl-2-oleoyl-sn-glycero-3-phosphatidylethanolamine (POPE), 1-palmitoyl-2-oleoyl-sn-glycero-3-phosphoserine (POPS), 1-palmitoyl-2-oleoyl-sn-glycero-3-phosphoinositol-3-phosphate (PI-3P), N-stearoyl-D-erythro-sphingosylphosphorylcholine (PSM), sn-(3-oleoyl-2-hydroxy)-glycerol-1-phospho-sn-1’-(3’-oleoyl-2’-hydroxy)-glycerol (BMGP, bis(monoacylglycero)phosphate), and cholesterol (CHOL). For the first step (system 1), before the MD run, two FP molecules were minimized and equilibrated in water in the presence of 0.15 M NaCl (system 1-1) or 0.15 M CaCl_2_ (system 1-2) (Supplementary Table S1). After the 150 ns MD simulation run, the FP peptides were selected for the second step (system 2) where six different membrane systems were used. The bilayer systems were composed of one specific type of phospholipid plus cholesterol (CHOL). In our case, the membranes consisted of POPC/CHOL, POPE/CHOL, POPS/CHOL, PI-3P/CHOL, PSM/CHOL and BMGP/CHOL, each one containing 150 molecules of phospholipid and 50 molecules of sterol, i.e., a molar ratio of 3:1 (Supplementary Table S2). Each one of the membrane systems were built either in the presence of 0.15 M NaCl or in the presence of 0.15 M CaCl_2_ so that 12 different systems resulted (systems 2-1 to 2-6 and systems 2-7 to 2-12, Supplementary Table S2). For the second step the MD simulations were performed for 450 ns. For the third step (system 3), we have used a model biomembrane system having a similar lipid composition to the late endosomal membrane (Supplementary Table S3). Three independent membrane systems were assembled using the Charmm-Gui web server (http://www.charmm-gui.org (accessed on 2 December 2022), [43]). The model late endosomal membrane systems contained 68/69 molecules of POPC, 30 molecules of POPE, 8 molecules of POPS, 12 of PI-3P, 12 molecules of PSM, 23 molecules of BMGP and 36/37 of cholesterol (CHOL) [33,45,46] (Supplementary Table S3). Each one of the systems was composed of one molecule of FP, membrane bilayer, water, and NaCl at physiological conditions, i.e., a concentration of 0.15 M, enclosed in a rectangular box and a neutral setting (Supplementary Table S3) [47,48,49]. The FP peptide pertained to the averaged and minimized fifteen NMR structures (PDB code 7MY8 [19], see above). Each one of the FP peptides were placed in the membrane such as its centre of mass coincided with the centre of mass of the phosphate atoms of the phospholipids (see [19] for a proposed insertion of the FP in a membrane). Since the FP peptide was inserted in the upper monolayer, the lipids touching the protein were eliminated and therefore the upper monolayer contained less lipids than the lower one (Supplementary Table S3). With respect to phospholipids, the palmitoyl chain is saturated, whereas the oleoyl chain contains a cis double bond, the presence of which increases the fluidity of the acyl chains of the phospholipids. All systems contained excess water [44].

VMD software (version 1.9.4 (University of Illinois, Urbana-Champaign, IL, USA)) was used for analysis and visualization [41,42,50,51]. The secondary structure of the peptides, the number of molecular contacts and the number of hydrogen bonds were obtained using VMD plugins [42]. S_CD_ order parameters, membrane thickness, molecular areas, and centre-of-mass were obtained using the VMD script collection “Membplugin” [42,51]. Hydrogen bonds were defined by a distance less than 3 Å between acceptor and donor atoms and an acceptor-H-donor angle of at least 150° [52]. The hydrogen bond interaction patterns and hydrophobic contacts between the phospholipids and the FP amino acids have been obtained using LigPlot+ (version 2.2 (European Bioinformatics Institute, Cambridge, UK)) [53]. Structural alignment of peptides has been obtained by using Mustang [54]. The complete simulations were used for the analysis unless otherwise indicated.

## 3. Results

In order to observe the binding of the FP peptide to a specific phospholipid in the membrane, knowing that both FP1 and FP2 segments are necessary as well—as it seems that Ca^2+^ ions might be required or at least increase the fusion rate—as a first step we ran a 150 ns MD simulation of the whole FP structure in water (PDB code 7MY8 [19]) in the presence of either NaCl or CaCl_2_ [19,20,25,26]. The systems were run for 150 ns in the presence of 0.15 M NaCl (system 1-1) or in the presence of 0.15 M CaCl_2_ (system 1-2). Each one of the systems contained two FP peptides (Supplementary Table S1) and therefore at the end of the MD simulation we obtained four FP different structures, two in the presence of NaCl (Figure 1B,C) and two in the presence of CaCl_2_ (Figure 1D,E). The superposition of the four final FP structures is shown in Figure 1F. The overall structure of the FP at the beginning of the MD simulation was 55% α-helix, 14% 3_10_-helix, 14% coil and 17% turns (rounded to the first integer). After 150 ns of simulation and in the presence of NaCl, one of the FP peptides presented an overall secondary structure of 49% α-helix, 29% coil and 21% turns whereas the other one presented an overall secondary structure of 29% α-helix, 27% coil and 44% turns. Similarly, for the system containing CaCl_2_, the overall secondary structure for one of the peptides was 45% α-helix, 29% coil and 26% turns whereas the other one presented an overall secondary structure of 50% α-helix, 30% coil and 20% turns. The secondary structure of the two FP peptides in the presence of CaCl_2_ was similar; however, in the presence of NaCl they were to some extent different.

**Figure 2:**
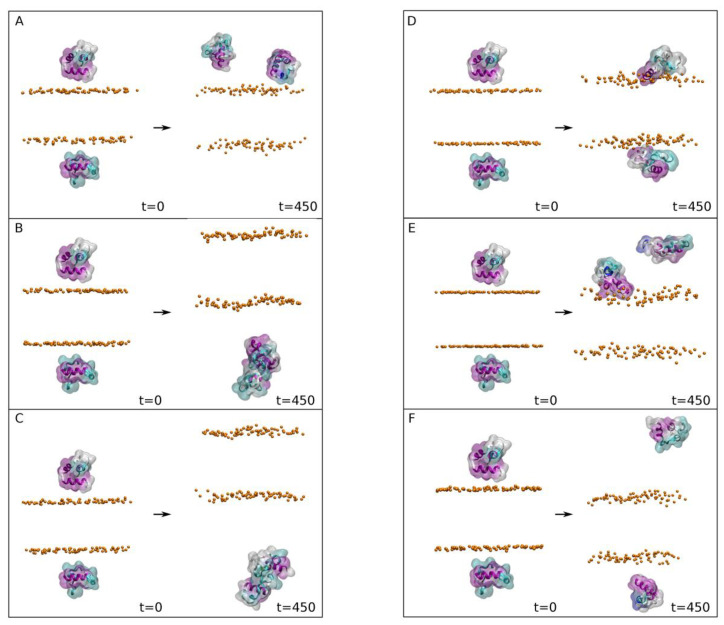
Initial (left) and final (right) arrangements corresponding to the second step (systems 2-1 to 2-6) for systems containing (**A**) POPC/CHOL, (**B**) POPE/CHOL, (**C**) POPS/CHOL, (**D**) PI-3P/CHOL, (**E**) BMGP/CHOL and (**F**) PSM/CHOL in the presence of 0.15 M NaCl (MD time 450 ns). The peptides are shown in both ribbon and surface transparent drawing style with coloured secondary structures (white (coil), violet (helix), yellow (extended), light blue (turns)). The surrounding phospholipid phosphate atoms are shown in VDW drawing style (orange). The water molecules and the chloride and sodium ions have been removed for clarity.

**Figure 3:**
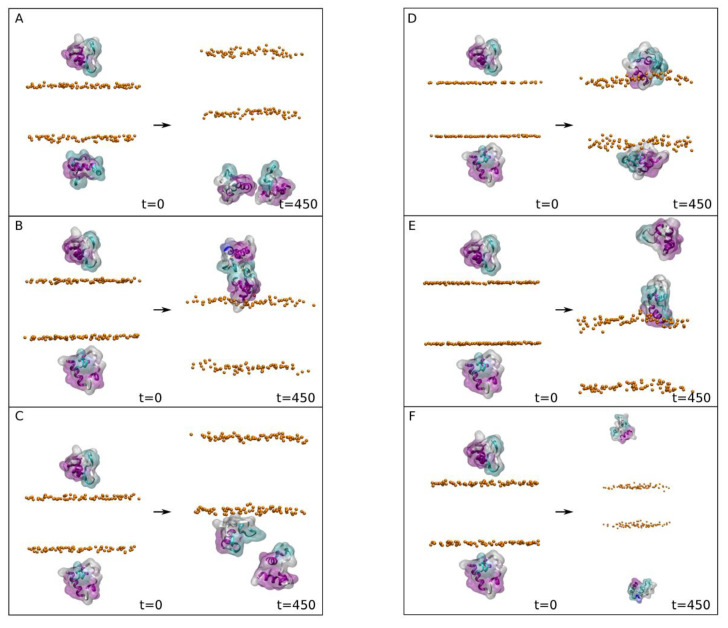
Initial (left) and final (right) arrangements corresponding to the second step (systems 2-7 to 2-12) for systems containing (**A**) POPC/CHOL, (**B**) POPE/CHOL, (**C**) POPS/CHOL, (**D**) PI-3P/CHOL, (**E**) BMGP/CHOL and (**F**) PSM/CHOL in the presence of 0.15 M CaCl_2_ (MD time 450 ns). The peptides are shown in both ribbon and surface transparent drawing style with coloured secondary structures (white (coil), violet (helix), yellow (extended), light blue (turns)). The surrounding phospholipid phosphate atoms are shown in VDW drawing style (orange). The water molecules and the chloride and calcium ions have been removed for clarity.

We are aware that our membrane systems consisting of a single type of phospholipid plus cholesterol are artificial since they do not represent a biological membrane. However, and as noted above, we aimed to observe any preferential binding of the FP to one and only one specific type of phospholipid. Therefore, and for the second MD step, we built six different biomembrane systems, each one consisting of one type of phospholipid plus cholesterol. Furthermore, each one of the bilayer systems were placed in two different types of environment, one with NaCl and the other with CaCl_2_. The four FP structures obtained in the first step were used for the second one since the secondary structure of the FP peptides were slightly different when subjected to MD in water. The membranes which were used were composed of POPC/CHOL, POPE/CHOL, POPS/CHOL, PI-3P/CHOL, PSM/CHOL and BMGP/CHOL, each one containing 150 molecules of phospholipid and 50 molecules of sterol, 100 lipids per leaflet, with a molar ratio of 3:1. It should be recalled that, in contrast to the plasma membrane, the late endosomal membrane is characterized by the high content of the negatively charged phospholipids PI-3P and BMGP [33]. The membranes were built either in the presence of 0.15 M NaCl (systems 2-1 to 2-6, Figure 2) or in the presence of 0.15M CaCl_2_ (systems 2-7 to 2-12, Figure 3); in the end, 12 different systems resulted (Supplementary Table S2). At the beginning, the average distance between the surface of the FP peptides and the membrane surface defined by the phosphate atoms of the phospholipid headgroups was about 10 Å (Figure 2 and Figure 3, t = 0 ns). This second step of the MD simulations was run for a total of 450 ns. For all the systems the membrane thickness remained fairly constant after ~10 ns (not shown for briefness), indicating that the membrane systems were completely equilibrated [41]. The average membrane thickness (Supplementary Table S2) was comparable to those membranes systems containing CHOL [55].

We have obtained the average mass density for the last 20 ns for selected phospholipid atoms of the model systems (not shown for briefness). All profiles were essentially symmetric between the two leaflets of the membrane, implying a comparable behaviour for all the lipids inside them. As expected, the location of the oxygen atoms of CHOL was deeper than the phospholipid phosphate atoms but relatively close to them, and the phospholipid methyl groups were partially intermingled but there was no interdigitation of the hydrocarbon chains.

Analysing the data obtained for the different systems in the presence of NaCl (Figure 2), it is possible to observe that there were two systems where at least one of the peptides was found bound to the membrane surface at the end of the MD simulation (Figure 2D,E with 2 and 1 bound peptides, respectively). Two peptides in the system 2-4 were bound to the membrane surface in the PI-3P/CHOL system (system 2-4, Figure 2D), whereas only one was bound to the membrane surface in the BMGP/CHOL system (system 2-5, Figure 2E). This behaviour can be followed by observing the *z*-axis centre of mass (COM) of the peptides with respect to the membrane surface (defined by the *z*-axis COM of the phosphate atoms of the phospholipids) for all the systems in the presence of 0.15 M NaCl (Supplementary Figure S1). It is clearly observable that all the peptides, except those noted above, present a large variation in the *z*-axis COM, indicating that they are not bound to the membrane surface (Supplementary Figure S1A–C,E,F). However, the bound peptides present a nearly constant *z*-axis COM with respect the membrane surface from the beginning, indicating that they are bound to the membrane and likely will remain bound (Supplementary Figure S1D,E). This behaviour can be perfectly observed in Supplementary Figure S3A for the systems PI-3P/CHOL and BMGP/CHOL (system 2-4 and 2-5, respectively), where the average *z*-axis COM of the CA carbon atoms of the two peptides for the last 20 ns of MD simulation are shown. This is further corroborated by the data represented in Supplementary Figure S3B, where the global average separation from the *z*-axis COM of the CA carbon atoms of the two peptides to the membrane surface are represented. As observed in the figure, the shortest global separation is found for the system PI-3P/CHOL (system 2-4), followed by system BMGP/CHOL (system 2-5). The largest separation was found for systems POPS/CHOL (system 2-3) and PSM/CHOL (system 2-6), whereas those of systems POPC/CHOL (system 2-1) and POPE/CHOL (system 2-2) were intermediate between the other ones (Supplementary Figure S3B). These data would imply that the FP, in the presence of NaCl, would bind with a great affinity to PI-3P and BMGP phospholipid containing membranes.

Analysing the data obtained for the different systems in the presence of CaCl_2_ (Figure 3), it is possible to observe that there were four systems where at least one of the peptides was found bound to the membrane surface at the end of the MD simulation (Figure 3B–E, with 1, 1, 2 and 1 bound peptides, respectively). Therefore, and in principle, the presence of CaCl_2_ increases the probability of the FP peptide to be bound to the membrane surface in comparison to the presence of NaCl. This behaviour can be clearly observed by examining the *z*-axis centre of mass (COM) of the peptides with respect to the membrane surface (defined by the *z*-axis COM of the phosphate atoms of the phospholipids) for all the systems in the presence of 0.15 M CaCl_2_ (Supplementary Figure S2). In this case there are two systems where the two FP peptides fluctuate in the water solvent, never touching the membrane surface, i.e., systems composed of POPC/CHOL (system 2-7) and PSM/CHOL (system 2-12) (Supplementary Figure S2A,F, respectively). In contrast there were three systems where only one FP peptide of the two present in the system was bound to the membrane surface at the end of the MD simulation. These systems were POPE/CHOL (system 2-8), POPS/CHOL (system 2-9) and BMGP/CHOL (system 2-11) (Supplementary Figure S2B,C,E, respectively). Finally, there was one system where the two peptides were found to be bound to the membrane surface, which was the system composed of PI-3P/CHOL (system 2-10) (Supplementary Figure S2D). For all those peptides which were found to be bound to the membrane surface their *z*-axis COM distance was relatively constant through the MD simulation (Supplementary Figure S2). This behaviour can be perfectly observed in Supplementary Figure S3A for the systems POPE/CHOL (system 2-8), POPS/CHOL (system 2-9), BMGP/CHOL (system 2-11) and PI-3P/CHOL (system 2-10), where the average *z*-axis COM of the CA carbon atoms of the peptides for the last 20 ns of MD simulation are shown. This is further corroborated by the data represented in Supplementary Figure S3C, where the global average separation from the *z*-axis COM of the CA carbon atoms of the two peptides to the membrane surface are represented. As observed in the figure, the shortest global separation is found for the system PI-3P/CHOL (system 2-10). In this case, this result is similar to that found previously in the presence of NaCl. The global separation for the systems composed of POPS/CHOL (system 2-9), POPE/CHOL (system 2-8) and BMGP/CHOL (system 2-11) was slightly higher that the system PI-3P/CHOL whereas the global separation of the systems POPC/CHOL (system 2-7) and PSM/CHOL (system 2-12) was significantly higher (Supplementary Figure S3C). These data would imply that the FP, in the presence of 0.15 mM CaCl_2_, would bind with a great affinity to the PI-3P phospholipids, with a lower affinity to POPE, POPS and BMGP, and none at all to POPC and PSM.

The average relative *z*-axis distances for the CA carbon atoms of each one of the residues in the membrane-bound peptides (see above) are shown in Supplementary Figure S4. Two different segments can be observed to be bound preferentially to the membrane, i.e., segments ^825^KVTLADAG^832^ (Supplementary Figure S4B,C,E,H) and ^835^KQYGDCL^841^ (Supplementary Figure S4A,F). It can also be observed that the overall structure of the FP is maintained for nearly all systems, independently of the system (see below). Since the overall proximity to the membrane surface gives us an idea of the capacity of the FP to be bound to a specific membrane, we have plotted the cumulative sum of the relative distances of all peptide Cα carbons for each system to the membrane surface, with the results are shown in Supplementary Figure S4I (the lower number, the tighter bound peptide). It can be observed in the figure that both PI-3P and BMGP phospholipids are the ones which the FP preferentially bind. By observing the sequence of the two peptides segments commented above, the existence of a Lys residue is apparent. Since one of the most important characteristics of PI-3P and BMGP phospholipids is their negatively charged headgroup, it can be implicitly supposed that the most important binding force should be of an electrostatic nature. However, the Lys residues in the FP sequence are surrounded by several hydrophobic amino acids, namely Leu, Phe, Val, Ile and Ala residues (Figure 1A), which imply that these hydrophobic residues might be the driving force to interact with the membrane, but the Lys residues are the ones which bind the PI-3P and BMGP headgroups (see below).

We have chosen the eight systems which showed stronger and more stable interaction of the FP with the membrane surface in order to obtain the binding characteristics between the FP peptide and the phospholipids (systems 2-4, 2-5, 2-8, 2-9, 2-10 and 2-11, Supplementary Figure S3). In the first place we have obtained the average number of contacts between the FP and each of the lipids in the systems for the last 20 ns of simulation (Supplementary Table S4). For systems 2-4 and 2-10, i.e., those systems containing membranes composed of PI-3P/CHOL in the presence of either NaCl or CaCl_2_, respectively, there was a considerable number of contacts (Supplementary Table S4). However, contacts were established almost exclusively with the phospholipid PI-3P but not with CHOL. The number of PI-3P molecules surrounding the FPs at a distance lower than 5 Å in the system 2-4 were 15 and 16 and in the system 2-10 were 12 and 18. However, the number of CHOL molecules surrounding the FPs in the system 2-4 was 1 and 0 and in the system 2-10 was 0 and 3. For systems 2-5 and 2-11, i.e., those systems containing membranes composed of BMGP/CHOL in the presence of either NaCl or CaCl_2_, respectively, there was a significant number of contacts, but fewer than those found for systems composed of PI-3P/CHOL (Supplementary Table S4). The larger number of contacts was found between the FPs and the phospholipid BMGP, but a small number was also found between the peptide and CHOL. The number of BMGP molecules surrounding the FPs in the system 2-5 was 11 and in the system 2-11 was 12. This is in contrast with the number of CHOL molecules surrounding the FPs, since in the system 2-5 there were two and in the system 2-11 there was one. For system 2-8, i.e., systems containing membranes composed of POPE/CHOL in the presence of CaCl_2_, there was a significant number of contacts but fewer than those found previously for systems PI-3P/CHOL and BMGP/CHOL (Supplementary Table S4). In this case the peptide only presented any contact with the phospholipid but not with the CHOL molecule. The number of POPE and CHOL molecules surrounding the FP in this system was 9 and 1, respectively. For system 2-9, i.e., systems containing membranes composed of POPS/CHOL in the presence of CaCl_2_, there was a lower number of contacts, much lower than those found previously for the other systems (Supplementary Table S4). In this case the peptide only presented any contact with the phospholipid but not with the CHOL molecule. The number of POPS and CHOL molecules surrounding the FP in this system was 10 and zero, respectively. We have also measured the number of hydrogen bonds between the FP and the lipids in these systems (Supplementary Table S4). The data shows that only PI-3P is capable of establishing hydrogen bonds with the peptide and, significantly, the number of hydrogen bonds between the two molecules is about the same in both the presence of NaCl or CaCl_2_. The phospholipids BMGP, POPE and POPS did not establish any hydrogen bond between them and the FP. Supplementary Figure S5 shows the final structures of the FP peptides including the lipids surrounding them. As observed in the figure, it can be observed that the FP is more strongly inserted into the membrane systems containing PI-3P and BMGP, much more with the first than with the second one (Supplementary Figure S5A–D for the former and Supplementary Figure S5E,F for the later). However, the FP peptides are loosely attached to the membranes composed of POPE and POPS (Supplementary Figure S5G,H, respectively). We have found that CHOL does not establish any hydrogen bonds with the FP and the number of contacts is very scarce in all the systems we have studied in this work, in contrast to other reports which describe the possibilities [56]. This is something which could be foreseen due to the nearly null number of CHOL molecules surrounding the FP. However, there were a high number of BMGP molecules surrounding the FP, yet a small number of contacts and no hydrogen bonds were observed.

We have measured the percentage of secondary structure for the FP in systems 2-4 and 2-10 (PI-3P-containing systems in the presence of NaCl and CaCl_2_, respectively) and systems 2-5 and 2-11 (BMGP-containing systems in the presence of NaCl and CaCl_2_, respectively) for the last 20 ns of MD simulation. The average dp structure for systems 2-4 was 34% α-helix, 42% coil and 24% turns, whereas for the FP in systems 2-10 it was 37% α-helix, 31% coil and 32% turns. For systems 2-5 the average FP structure it was 38% α-helix, 33% coil, 9% 3_10_-helix and 19% turns, whereas for the FP in systems 2-11 it was 22% α-helix, 40% coil and 38% turns. These secondary structure percentages were significantly different from the percentages of the initial structure (PDB code 7MY8 [19]), but they were relatively similar to the secondary structure percentages which were found after 150 ns of simulation in water (see above). Although there are differences between the different peptides, these differences are not very significant, so it can be said that the global structure of the FP peptides after 450 s do not change dramatically upon binding to different phospholipid types in the presence of both types of ions, Na^+^ or Ca^2+^. In the systems containing Ca^2+^ this ion was preferably bound to the acidic amino acids of the FP along the last ns of MD simulation (not shown for briefness), but its binding did not preclude the binding of the FP to the membrane.

Molecules interacting with the surface and/or the interior of the membrane influence the hydrocarbon chain order of the phospholipid acyl chains. Therefore, we have explored the effect of the FP on the hydrocarbon chain order analysing their deuterium order parameter, S_CD_ (Supplementary Figure S6). For complete disorder of the hydrocarbon chains, the S_CD_ value is 0 but 0.5 for full order along the normal bilayer [57]. The bulk average −S_CD_ values of the hydrocarbon chains of all phospholipids in the different membrane systems agreed with the data observed previously for experimental and simulated data [40,58,59] (Supplementary Figure S6). Nevertheless, there were significant, although not impressive, changes on the S_CD_ profiles for some of the phospholipids near the FP molecules. The most considerable effect was observed for the hydrocarbon chains of PI-3P molecules surrounding the FP, since a general decrease in the S_CD_ values was observed, larger for the oleoyl chain than the palmitoyl one (Supplementary Figure S6A–D). It should be remembered that the oleoyl hydrocarbon chain is more flexible than the palmitoyl chain so that the former can adapt in a better way to the proximity of the FP than the later one. There was also a significant effect on the hydrocarbon chains of FP surrounding BMGP phospholipids since, as commented above, a general decrease in the S_CD_ values was observed (Supplementary Figure S6E–H). However, the observed effect was smaller than the one observed for PI-3P. For POPE and POPS phospholipids surrounding the FP there was also a decrease observed in the S_CD_ values, more apparent in the oleoyl the in the palmitoyl chains, but lower than for PI-3P and BMGP (Supplementary Figure S6I,J for POPE and 6K,L for POPS). The decrease in the S_CD_ values observed for the phospholipids surrounding the FP indicates that the FP increases the fluidity of the hydrocarbon chains of these phospholipids. However, the fluidity effect is different for each type of lipid, being bigger for PI-3P and BMGP than POPE and POPS (i.e., PI-3P > BMGP > POPE ~ POPS). Although the FP inserts into the interfacial zone of the bilayer and not into the hydrocarbon palisade structure, it affects its fluidity, with a more striking effect on PI-3P and BMGP. This data would corroborate what has been commented above, i.e., that the FP tends to preferably bind the electronegative phospholipids, with preference for PI-3P rather than BMGP.

**Figure 4:**
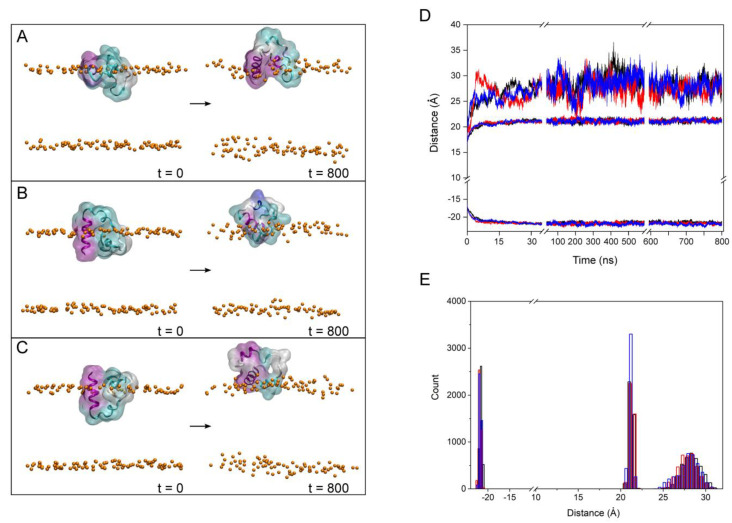
Initial (t = 0 ns) and final (t = 800 ns) arrangements corresponding to the third step, i.e., model membrane similar to the late endosomal one, for (**A**) system 3-1, (**B**) system 3-2 and (**C**) system 3-3 in the presence of 0.15 M NaCl (MD time 800 ns). The peptides are shown in both ribbon and surface transparent drawing style with coloured secondary structures (white (coil), violet (helix), yellow (extended), light blue (turns)). The phospholipid phosphate atoms are shown in VDW drawing style (orange) and the water molecules. The chloride and sodium ions have been removed for clarity. (**D**) Time variation of the *z*-axis distance (centre of the membrane as reference) and (**E**) distance histograms of the FP peptide and the phosphate atoms of the phospholipids (systems 3-1 (black), 3-2 (red) and 3-3 (blue), last 30 ns of the MD simulation).

As we commented above, we are aware that the membrane systems which consist of a single type of phospholipid plus CHOL do not represent a biological membrane. However, they give us a very good idea of the propensity of the FP to bind and specifically interact with a phospholipid in the membrane as we have shown above. Taking this fact into account, we have further studied three independent model biomembrane systems that have a similar lipid composition to the late endosomal membrane (Supplementary Table S3), i.e., a membrane composed of POPC, POPE, POPS, PI-3P, PSM, BMGP and CHOL [33,45,46]. Moreover, the FP peptides were placed at the same relative location with respect to the centre of mass of the phosphate atoms of the phospholipids [19], but with three different orientations (Figure 4A–C, t = 0 ns). This third step of the MD simulations was run for a total of 800 ns. For all the three systems, membrane thickness and lipid areas remained fairly constant after ~35 ns (not shown for briefness), indicating that the membrane systems were completely equilibrated after that time [41]. The average membrane thickness and the average lipid areas at the end of the MD simulation (Supplementary Table S3) were comparable to those membrane systems containing CHOL [55,60,61]. It should be taken into account that the interaction of peptides with membranes depends notably on bilayer thickness [62] and, in turn, bilayer thickness depends on the phospholipid types, their hydrocarbon chains and CHOL content [63]. The final snapshots for the three systems are also shown in the Figure 4A–C, t = 800 ns, where, significantly, it can be observed that the final location of the peptides is the same in all three systems. To observe the movement of the FP peptides in the systems during the whole simulation, we have obtained the time variation of the centre-of-mass (COM) of the FP peptides and compared them with the COMs of the phosphate atoms at both monolayers, which define the membrane surface (Figure 4D). As has been commented above, the COM values of the FPs and the phosphate atoms of the phospholipids were identical at the beginning of the MD simulation (Figure 4D). However, the COM values of the FPs moved rapidly out of the interface to remain adjoined to the membrane surface till the end of the simulation, without wandering out of it (Figure 4D). The histogram data for the last 30 ns reveal that their location was very stable, very similar and did not wander at all (Figure 4E). The average positions for the last 30 ns of the FP peptides for systems 3-1, 3-2 and 3-3 were 28.3 ± 1.1 Å, 27.8 ± 1.1 Å and 27.7 ± 1.3 Å, respectively (Figure 4E). That it is to say, the location of the FP peptides in the three systems were nearly identical at the end of the MD simulation and they were attached to the membrane (Figure 4A–C at t = 800 ns).

We have obtained the average mass densities of all the membrane lipids over the last 30 ns for systems (not shown for reasons of space). Between the two monolayers of the membrane, all contours are basically symmetric, which imply an equivalent behaviour for all the lipids in the membrane for the three model membrane systems. The same happens with the phosphate atoms of the phospholipids as well as with the oxygen atoms of CHOL, which are present at the same relative distance in the two systems studied (Supplementary Figure S7A–C). The mass density of the FP peptides extends to the interfacial part of the membrane as well as to the internal part, going across the phosphate atom layer to the area where the oxygen of the cholesterol molecule is located, but never reaching beyond (Supplementary Figure S7A–C). Inspecting the mass density profile of the FP peptides, their location is very well defined, but their overall form is not, meaning that their relative orientation is different (compare dotted lines in Supplementary Figure S7A–C). The average relative *z*-axis distances for the CA carbon atoms of each one of the residues in the membrane-bound FP peptides are shown in Supplementary Figure S7D. Similarly to what has been found above, two different segments can be observed to be bound preferentially to the membrane, i.e., segments ^825^KVTLADAGFIKQY^837^ and ^844^VAARDLIC^851^, which approximately match each one of the conserved FP1 and FP2 sequences of the spike S protein [12,15,16,20] (see above). These and the data presented above would imply that segments FP1 and FP2, which together make up the FP, can bind the membrane independently of each other. Supplementary Figure S7E shows the average percentage of the secondary structure of the FP peptides for the first and the last 30 ns of MD simulation. Although the standard deviation is relatively high, considering that the average has been taken for three independent systems for 30 ns of simulation time, the data demonstrate that the overall secondary structure of the peptides is maintained for the whole simulation time, i.e., 800 ns. 

We have also analysed the effect of the FP on the hydrocarbon chain order of the phospholipid acyl chains, with the results are shown in Supplementary Figure S8. We must remember that in this case we are analysing a complete membrane composed of six different phospholipids resembling the late endosomal one. The average bulk average –S_CD_ values of the hydrocarbon chains of all phospholipids agree with the data observed previously for experimental and simulated data [40,58,59]. Contrary to what was previously observed, the effect of the FP peptide in the nearby lipids is a very dramatic one since a significant decrease in the S_CD_ values can be observed affecting to all the phospholipids (Supplementary Figure S8). The decrease in the S_CD_ values observed for the phospholipids surrounding the FP would indicate that the peptide increases the fluidity of the hydrocarbon chains of these phospholipids. In this case, the decrease in the S_CD_ values was observed for both oleoyl and palmitoyl chains, indicating that the peptide affects the entire phospholipid molecule and everyone near it.

We obtained the average number of contacts between the FP and each of the lipid in the late endosomal membrane systems for the last 30 ns of simulation (Supplementary Table S5). Comparing the contacts between the FP and all the lipids with those of the major lipid in the membrane, i.e., POPC, the number of contacts is significantly higher for BMGP than expected, as well as slightly higher for POPS and PSM. On the contrary, it is significantly lower for PI-3P, followed by POPE and CHOL. If we look at the data corresponding to the number of lipids around the FP peptides, and likewise compare them with POPC, we can see that they roughly correspond to the relative number of them in the upper layer of the membrane where the FP resides. Therefore, there are no significant differences in the number of lipids surrounding the FP in any of the three systems studied. We also obtained the average number of hydrogen bonds between the lipids and the FP, but there were practically none between them.

**Figure 5:**
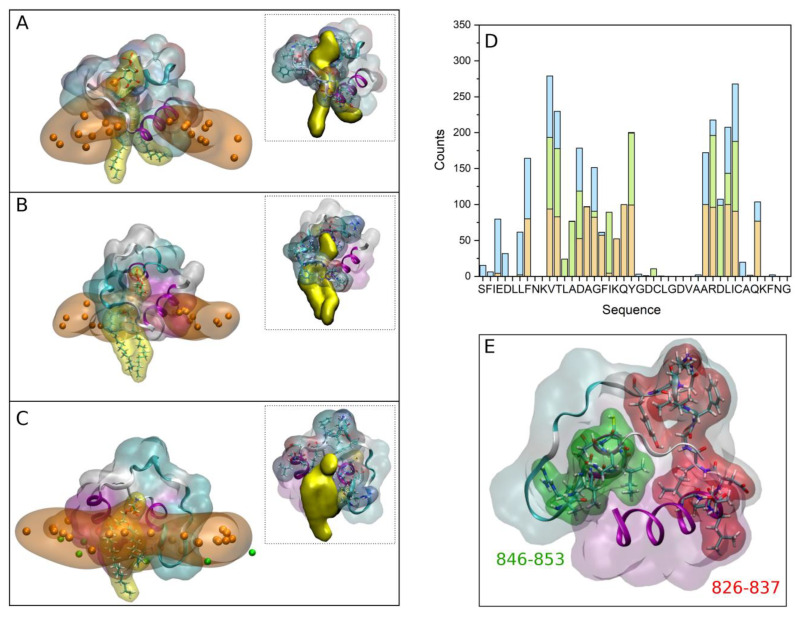
Final composition of the FP with the surrounding phosphate atoms at a distance of 10 Å or less for (**A**) system 2-4, (**B**) system 2-10 and (**C**) system 3-1. The FP is shown in ribbons and transparent surface drawing, whereas the phosphate atoms of the phospholipids are shown in VDW and transparent orange surface drawing. The phospholipid intimately bound to the FP is shown in licorize and yellow transparent surface drawing (PI-3P in (**A**, **B**) and POPS in (**C**)). The inserts in (**A**–**C**) show the tight association of the FP (ribbons and transparent surface drawing) and the phospholipid (yellow opaque surface drawing), highlighting the residues in contact with the lipid (licorize drawing). (**D**) Histogram of the average of contacts per residue between the FP and the phospholipids in (**A**–**C**) for the last 30 ns (orange, green and blue, respectively). (**E**) Structure of the FP showing the two binding regions (licorize drawing) observed in (**D**). The FPs are shown with coloured secondary structures (white (coil), violet (helix), yellow (extended), light blue (turns). The oxygen atoms of CHOL are shown in VDW green colour in panel (**C**).

As previously commented, those systems containing PI-3P were the ones that showed a stronger binding of the FP peptides to the membrane when they were composed of one type of phospholipid plus CHOL. Moreover, they were the only ones which showed the presence of hydrogen bonds between the peptides and the lipids (cf. Supplementary Table S4). We have also studied the interaction of the FP with a model membrane similar to the late endosomal one, that is, containing a complex mixture of phospholipids plus CHOL, observing that the peptide remains attached and interacting with it. In this case we have noticed that in one of the systems, system 3-1, there was a tight binding between the FP and a phospholipid, POPS (check small density band at ~28 Å in Supplementary Figure S7A). A detailed picture of the arrangement of the FP in each one of the membranes showing the tight binding to the phospholipids is shown in Figure 5A for system 2-4, Figure 5B for system 2-10 and Figure 5C for system 3-1 (the phosphate atoms of the phospholipids demarcate the membrane surface). For each one of the arrangements there is one and only one phospholipid wrapped by the peptide, protruding from the membrane and in a tight association with the peptide. The tight association between the FP and the phospholipids is perfectly observed in the inserts of Figure 5A–C where each one of the FP and the phospholipid molecules are displayed in a single colour. For each one of those three systems we have calculated the number of contacts for the last 30 ns of the MD simulation, with the results are presented in Figure 5D (the amino acid residues capable of presenting hydrogen bonds with the phospholipid are very few, but the number of amino acids that can present contacts is much greater). As observed in the figure, two specific regions stand out in the sequence, the region covering residues from 826 to 837, ^826^VTLADAGFIKQY^837^, and the region covering residues from 846 to 853, ^846^ARDLICAQ^853^ (Figure 5D). The first of these regions encompasses part of the FP1 sequence and part of the FP2 sequence, while the second is entirely contained in FP2. We can highlight in the first region the basic and acidic amino acids ^835^Lys and ^830^Asp as well as the hydrophobic ones ^828^Leu and ^834^Ile, whereas in the second one we can highlight the basic and acidic amino acids ^847^Arg and ^848^Asp as well as the hydrophobic ones ^849^Leu and ^850^Ile (Figure 5D). The existence of basic and acidic residues, as well as hydrophobic residues, must be responsible for such a strong interaction between the FP and the negatively-charged phospholipids. The two regions commented above are highlighted in the secondary structure of the FP shown in Figure 5E. As observed in the figure, the two regions are separated by a stretch of eight amino acids giving them some flexibility. It could be thought that the set of the two aforementioned regions plus the relatively flexible zone in between them could form a clamp with which to encompass the negatively-charged phospholipids (see inserts in Figure S5A–C). Since one of the most important characteristics of the phospholipids which bind to the FP is their negatively charged headgroup, it can be implicitly supposed that the most important binding force should be of an electrostatic nature. However, the basic residues in the FP sequence are surrounded by several hydrophobic amino acids, namely Leu, Phe, Val, Ile and Ala residues (Figure 1A and Figure 5D), which imply that these hydrophobic residues might be the driving force to interact with the membrane, but the basic amino acids are the ones which bind the phospholipid headgroups.

## 4. Discussion

Cell entry of enveloped viruses and specifically SARS-CoV-2 can occur in two different ways, either through the plasma membrane or through the endosomal membrane or both [2,3,4,5]. The essential mechanism by which these viruses enter the host cell is called membrane fusion and it is one of the main targets for the development of novel antiviral therapies. The SARS-CoV-2 membrane fusion protein is the spike S class I membrane fusion protein, responsible for binding and entering into the cell through the S1 and S2 subunits, respectively [2,4,5,6,7]. The S2 subunit contains the fusion domain (FP) which has an essential role in the fusion between the viral and host membranes [4,7,9,10]. However, the membrane fusion mechanism by which the FP attaches and inserts into the membrane is not understood yet. Although it is known that the FP modulates the membrane biophysical properties, the structure of the FP when it binds to the membrane and the lipid specificity of binding is not known. The understanding of the membrane fusion mechanism and the specific interactions at the molecular level which take place in the membrane, being one of the most significant targets, will result in the effective development of effective antiviral strategies which will make possible the inhibition of the viral-cell interaction. With that aim, in this work we have used MD to study the binding and interaction of the SARS-CoV-2 FP to specific phospholipids in a model membrane.

We first studied the structure of the FP in water and in the presence of either Na^+^ or Ca^2+^ ions. At the end of the simulation, we obtained four different structures, not dramatically different from each other, such that we used the four of them for the next MD step, i.e., the FP peptide plus model membranes. These model biomembranes contained one type of phospholipid plus CHOL with a phospholipid/CHOL ratio of 3:1. The phospholipids used were POPC, POPE, POPS, PI-3P, PSM and BMGP and since the model membranes were studied in the presence of sodium and calcium, in the end twelve different systems were obtained, six in the presence of NaCl and six in the presence of CaCl_2_. After 450 ns of MD simulation, stronger interaction observed between the FP and the phospholipids was observed for PI-3P and BMGP, greater for the former than the later. The number of contacts between the FP and PI-3P is larger than those found between the FP and BMGP and, furthermore, the formation of hydrogen bonds is only found between the FP and PI-3P. The number of hydrogen bonds does not depend on the presence of either NaCl or CaCl_2_. No other phospholipids, including BMGP, form hydrogen bonds with the FP. Significantly, CHOL present a very low number of contacts whereas it does not form any hydrogen bond with the FP. This specific effect of the FP on both PI-3P and BMGP is corroborated by the increase in the fluidity observed on the hydrocarbon chains of these two types of phospholipids, larger with PI-3P than with BMGP. We have further studied the interaction and binding of the FP to the membrane by studying three independent model biomembrane systems that had a similar lipid composition to the late endosomal membrane. In this way, these membranes contained the same phospholipid types previously studied. Significantly, after 800 ns of MD simulation, the FP peptides remained attached to the membrane interface in a very similar position, indicating their high propensity to remain membrane bound. In a similar way to what was found in the previous step, two different segments were observed to be most likely bound to the membrane, i.e., segments ^825^KVTLADAGFIKQY^837^ and ^844^VAARDLIC^851^, matching the FP1 and FP2 sequences of the spike S protein. All these data would imply that both FP1 and FP2 sequences can bind the membrane independently of each other. Furthermore, and comparing the number of contacts between the FP and all the lipids with those of POPC, the major lipid in the membrane, the number of contacts was significantly higher for BMGP but much lower for CHOL, showing that the FP would tend to bind preferentially negatively-charged phospholipids but would exclude CHOL. It is interesting to note that it has been described that the FP from SARS-CoV-2 possess two CHOL-recognition motifs, which could suggest that FP could bind with CHOL at some point in the fusion process [27]. It could be possible that the first step in the fusion process would be the specific interaction of the FP with certain types of phospholipids and subsequently interact with the deepest part of the membrane where cholesterol resides. Remarkably, the FP increased the fluidity of the hydrocarbon chains of all surrounding phospholipids in the late endosomal membranes; in a later step this could increase the probability of the interaction of the FP with CHOL [21].

The secondary structure of the FP did not change dramatically upon phospholipid binding, independently of the membrane model system or the ion, implying that the overall structure of the FP both in solution and bound to the membrane is very similar. Significantly, and for the PI-3P-only-containing membrane, two FP structures were able of wrapping one PI-3P molecule each, whereas for the late endosomal membrane one FP structure was able of wrapping a POPS molecule. For the first two systems, there is a patch of residues which have the highest probability of forming hydrogen bonding with PI-3P, i.e., amino acids ^835^Lys, ^836^Gln, ^837^Tyr, ^838^Gly, ^839^Asp, ^847^Arg and ^854^Lys, both basic and acidic. Of the study carried out on the FP tightly-bound phospholipids, two peptide regions stand out in the sequence, regions ^826^VTLADAGFIKQY^837^ and ^846^ARDLICAQ^853^ (Figure 5). These regions, which coincide with the peptide segments interacting with membrane surface in all membrane systems, are composed of both charged and hydrophobic residues and both types should be responsible for the interaction between the FP and the phospholipids [26]. Since one of the most important characteristics of the negatively-charged phospholipids is their charged headgroup, it can be implicitly supposed that the most important binding force should be of an electrostatic nature. However, that is not the only feature involved in the binding process since there are several hydrophobic amino acids surrounding the basic and acidic ones, i.e., Leu, Phe, Val, Ile and Ala residues, which can establish a large number of contacts with the phospholipid as well as cholesterol [21,26,27]. It can be concluded that the membrane driving force is a combination of charged and hydrophobic residues, dividing the task to interact with the membrane on the one hand and specifically interacting with the electronegatively charged phospholipids on the other. The global arrangement of the FP would maximize their interactions with the phospholipids in the membrane, both with the membrane interphase and with the hydrocarbon chains of the lipids.

In order to reduce the energy required to achieve fusion amongst the viral and cellular membranes a mixture of different properties of the molecules involved must act together. For example, the binding of phospholipid head-groups by specific residues of the protein, physical changes in the membrane modulated by protein binding, ionization changes in both protein amino acids and lipid head-groups and the formation of specific lipid-enriched domains. The conceivable formation of negatively-charged enriched domains of electronegatively-charged phospholipids would enable the different FP domains from the same or different oligomers to bind the membrane, allowing other domains of the spike S protein to interact and fold with the host membrane and succeed in the collapsing of the viral and cellular membranes. Subsequently, several oligomers, acting together, would greatly perturb an area of the membrane which would eventually cause membrane fusion. Advancing in our understanding of the mechanism of membrane fusion as well as the inter- and intramolecular interactions which might exist between proteins and lipids will lead us to the development of general antiviral therapies that will enable the fight against these types of viruses.

## 5. Conclusions

Cellular entry of SARS-CoV-2 occurs by membrane fusion, an essential mechanism and one of the main targets for the development of new antiviral therapies. The S2 subunit of the SARS-CoV-2 S membrane fusion protein contains the fusion domain responsible for binding and interacting with lipids in the membrane. We have used molecular dynamics to study the binding and interaction of the SARS-CoV-2 fusion domain with phospholipids in a model membrane. We have observed a specific interaction of the fusion domain with electronegatively charged phospholipids. There are both hydrophobic and charged amino acids involved in membrane binding, implying that the driving force for fusion must be a combination of hydrophobic and electrostatically different effects, allowing the close association of the FP with the membrane phospholipids. Binding of the fusion domain to specific phospholipids in the membrane allows for the possibility of formation of domains in the membrane facilitating the interaction of other domains of the SARS-CoV-2 S protein with the host membrane. In conclusion, the charge of protein amino acids and lipid headgroups, the hydrophobicity of specific amino acids, the binding to electronegatively charged phospholipids, and the tight and unique wrapping of the fusion domain should be the first and most important stage in membrane fusion that would end in the collapse of the viral and cellular membranes.

## Data Availability

Data available on request.

## Supplementary Materials

The following supporting information can be downloaded at: https://www.mdpi.com/article/10.3390/membranes13030344/s1, Figure S1: Time variation of the z-axis distance (center of the membrane as reference) for systems containing (A) POPC/CHOL, (B) POPE/CHOL, (C) POPS/CHOL, (D) PI-3P/CHOL, (E) BMGP/CHOL and (F) PSM/CHOL in the presence of 0.15 M NaCl (MD time 450 ns). The global z-axis distance of the two FP peptides in the system are depicted in blue and red line colours, whereas the z-axis distance of the phosphate atoms of the phospholipids, which the membrane surface, is depicted in black (upper and lower boundaries); Figure S2: Time variation of the z-axis distance (center of the membrane as a reference) for systems containing (A) POPC/CHOL, (B) POPE/CHOL, (C) POPS/CHOL, (D) PI-3P/CHOL, (E) BMGP/CHOL and (F) PSM/CHOL in the presence of 0.15 M CaCl_2_ (MD time 450 ns). The global z-axis distance of the two FP peptides in the system are depicted in blue and red line colours, whereas the z-axis distance of the phosphate atoms of the phospholipids, which define the membrane surface, is depicted in black (upper and lower boundaries); Figure S3: (A) Average z-axis distance for the CA carbon atoms of the two FP peptides in each one of the systems (-■- and -■- for peptides 1 and 2, respectively) in relation to the average z-axis distance for the phosphate atoms of the phospholipids (-●-). (B, C) Average z-axis distance separation for all the CA carbon atoms of the two FP peptides to the average z-axis distance for the phosphate atoms of the phospholipids as a reference in the presence of (B) 0.15 M NaCl or (C) 0.15 M CaCl_2_. The systems correspond to POPC/CHOL (2-1, 2-7), POPE/CHOL (2-2, 2-8), POPS/CHOL (2-3, 2-9), PI-3P/CHOL (2-4, 2-10), BMGP/CHOL (2-5, 2-11) and PSM/CHOL (2-6, 2-12) in the presence of either (2-1 to 2-6) 0.15 M NaCl or (2-7 to 2-12) 0.15 M CaCl_2_. The data represent the average for the last 20 ns of the MD simulation; Figure S4: Average relative z-axis distance for the CA carbon atoms for each one of the residues of the FP peptides which are bound to the membrane surface along the MD simulation. Each peptide belong to systems (A) 2-4, (B) 2-4, (C) 2-5, (D) 2-10, (E) 2-10, (F) 2-9, (G) 2-8 and (H) 2-11. (I) Sum of the relative CA atoms z-axis distance for the specified phospholipids in each system. The data represent the average for the last 20 ns of the MD simulation; Figure S5: Final composition of the FP peptides in the systems (A) 2-4, (B) 2-10, (C) 2-10, (D) 2-4, (E) 2-5, (F) 2-11, (G) 2-8 and (H) 2-9. The peptides are shown in ribbon and transparent surface drawing style with coloured secondary structures (white (coil), violet (helix), yellow (extended), light blue (turns)). The amino acid residues forming hydrogen bonds with the PI-3P phospholipid are shown in VDW style. The lipids surrounding the peptides at a distance of 5A or less are also shown in licorize drawing style. The phospholipid phosphate and the CHOL oxygen atoms are shown in VDW drawing style (orange and yellow, respectively). The other lipids, water an ions have been removed for clarity; Figure S6: Average deuterium order parameter –S_CD_ computed for the hydrocarbon acyl chains of the phospholipids for systems (A, B) 2.4, (C, D) 2.10, (E, F) 2.5, (G, H) 2.11, (I, J) 2.8, and (K, L) 2.9. (A, C, I, K) palmitoyl and (B, D, E, F, G. H, J, L) oleoyl acyl chains of (A, B, C, D) PI-3P, (E, F, G, H) BMGP, (I, J) POPE and (K, L) POPS. The data correspond to the bulk phospholipid acyl chains (-■-) and the phospholipid acyl chains within 5 Å of the FP (-■-). The analysis was carried out for the last 20 ns of simulation; Figure S7: Mass density profiles for the last 30 ns of the MD simulation for (A) system 3-1, (B) system 3-2 and (C) system 3-3. The mass density profiles correspond to the phosphate atoms of POPC (black), POPE (blue), POPS (red), PI-3P (orange), PSM (olive), BMGP (magenta), the oxygen atom of CHOL (wine) and the FP peptide (dotted black line). (D) Average relative z-axis distance for the CA carbon atoms for each one of the residues of the FP peptides (systems 3-1 (black), 3-2 (red) and 3-3 (blue) for the last 30 ns of the MD simulation (the dotted line represents the average COM of the phosphate atoms of the phospholipids). (E) Average percentage secondary structure for the first (■ black) and the last (● red) 30 ns of simulation; Figure S8: Average deuterium order parameter –S_CD_ computed for the hydrocarbon acyl chains of the phospholipids for systems 3-1, 3-2 and 3-3 over the last 30 ns of simulation. (A, C, E, G) oleoyl and (B, D, F, H) palmitoyl acyl chains of (A, B) POPC, (C, D) POPE, (E, F) POPS, (G, H) PI-3P, (I) 3- and (J) 3’-oleoyl chains of BMGP and (K) palmitoyl and (L) sphyngosyl acyl chains of PSM. The data correspond to the bulk phospholipid acyl chains (-■-) and the phospholipid acyl chains within 5 Å of the FP (-●-); Table S1: Systems and components used in the first step of this study (FP in water). The NaCl (system 1-1) and CaCl_2_ (system 1-2) concentration was 0.15 M. The MD production trajectories for each one of the systems were computed for 150 ns; Table S2: Systems and components used in the second step of this study (FP peptides in the presence of the membrane systems). The NaCl and CaCl_2_ concentration was 0.15M. Membrane thickness at the end of the MS simulation is also shown. The MD production trajectories for each one of the systems were computed for 450 ns; Table S3: Systems and components used in the third step of this study (FP peptides in the presence of a biological model membrane). The NaCl concentration was 0.15 M. Membrane thickness at the end of the simulation is also shown. The MD production trajectories for each one of the systems were computed for 800 ns; Table S4: Number of contacts and hydrogen bonds between the FPs with the lipids for the selected membrane systems (rounded to the whole number, two FP´s per system). The data represent the average of the last 20 ns of the MD simulation; Table S5: Number of lipids around the FP up to 5 Å and contacts between the FPs and lipids. The data represent the average for the three systems and for the last 30 ns of the MD simulation.

## Abbreviations

BMGP	Bis(monoacylglycero)phosphate [(sn-(3-oleoyl-2-hydroxy)-glycerol-1-phospho-sn-1’-(3’-oleoyl-2’-hydroxy)-glycerol)]
CHOL	cholesterol
COM	Centre of masses
FP	Fusion peptide
PI-3P	1-palmitoyl-2-oleoyl-sn-glycero-3-phosphoinositol-3-phosphate
POPC	1-palmitoyl-2-oleoyl-sn-glycero-3-phosphocholine
POPE	1-palmitoyl-2-oleoyl-sn-glycero-3-phosphoethanolamine
POPS	1-Palmitoyl-2-oleoyl-sn-glycero-3-phosphoserine
PSM	N-Stearoyl-D-erythro-sphingosylphosphorylcholine
