# Supplementary material for: SARS-CoV-2 Protein S Fusion Peptide Is Capable of Wrapping Negatively-Charged Phospholipids
Source: Membranes (Basel). 2023 Mar 16;13(3):344. doi: 10.3390/membranes13030344 (PMC10057416; doi:10.3390/membranes13030344)
Supplement: Supplementary file 1 [file membranes-13-00344-s001.zip › membranes-2181390-supplementary.pdf]

membranes

Supplementary Materials

SARS-CoV-2 Protein S Fusion Peptide Is Capable of Wrapping Negatively-Charged Phospholipids

José Villalaín

Institute of Research, Development, and Innovation in Healthcare Biotechnology (IDiBE), Universitas "Miguel Hernández", E-03202 Elche, Spain; jvillalain@umh.es; Tel.: +34-648-891-404

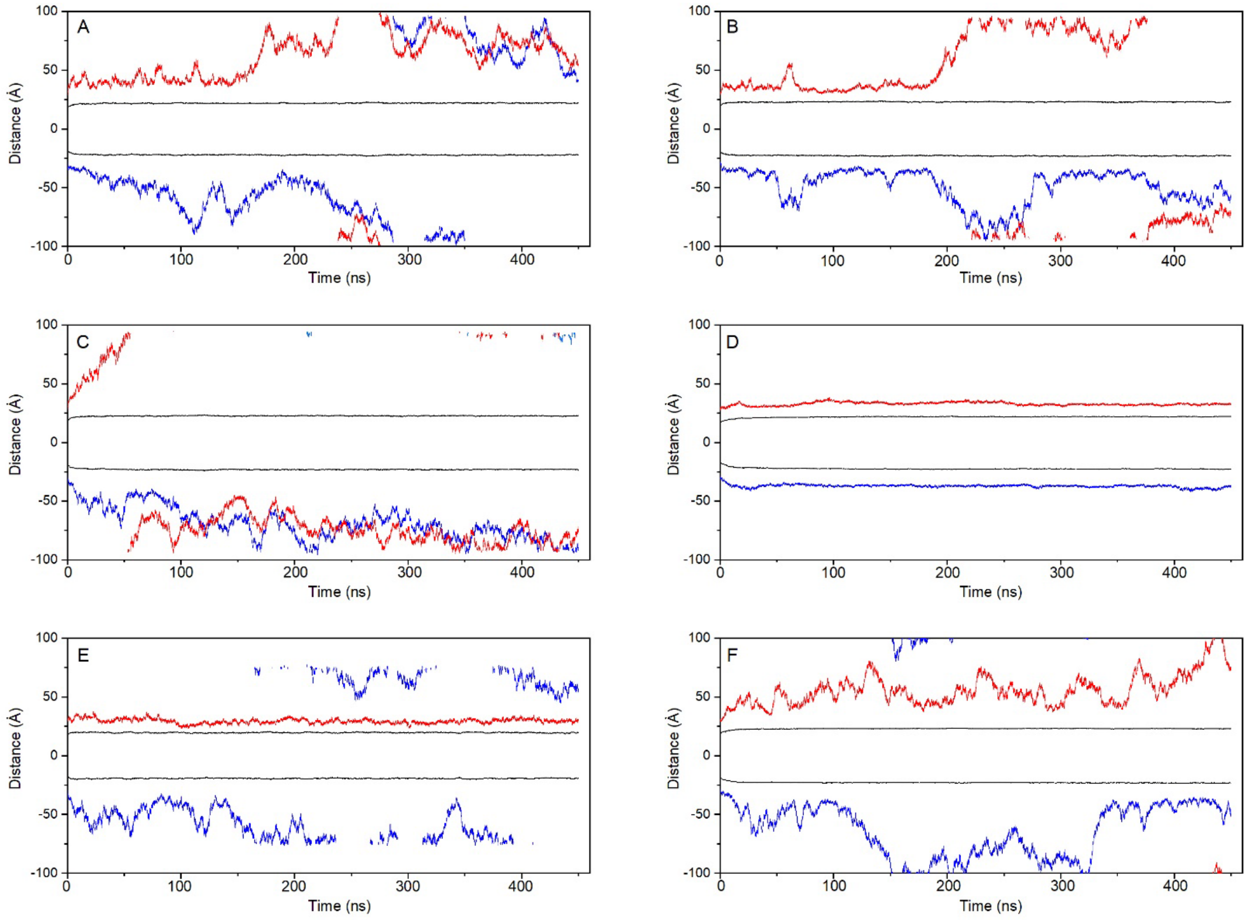

Figure S1. Time variation of the z-axis distance (center of the membrane as reference) for systems containing (**A**) POPC/CHOL, (**B**) POPE/CHOL, (**C**) POPS/CHOL, (**D**) PI-3P/CHOL, (**E**) BMGP/CHOL and (**F**) PSM/CHOL in the presence of 0.15 M NaCl (MD time 450 ns). The global z-axis distance of the two FP peptides in the system are depicted in blue and red line colours, whereas the z-axis distance of the phosphate atoms of the phospholipids, which the membrane surface, is depicted in black (upper and lower boundaries).

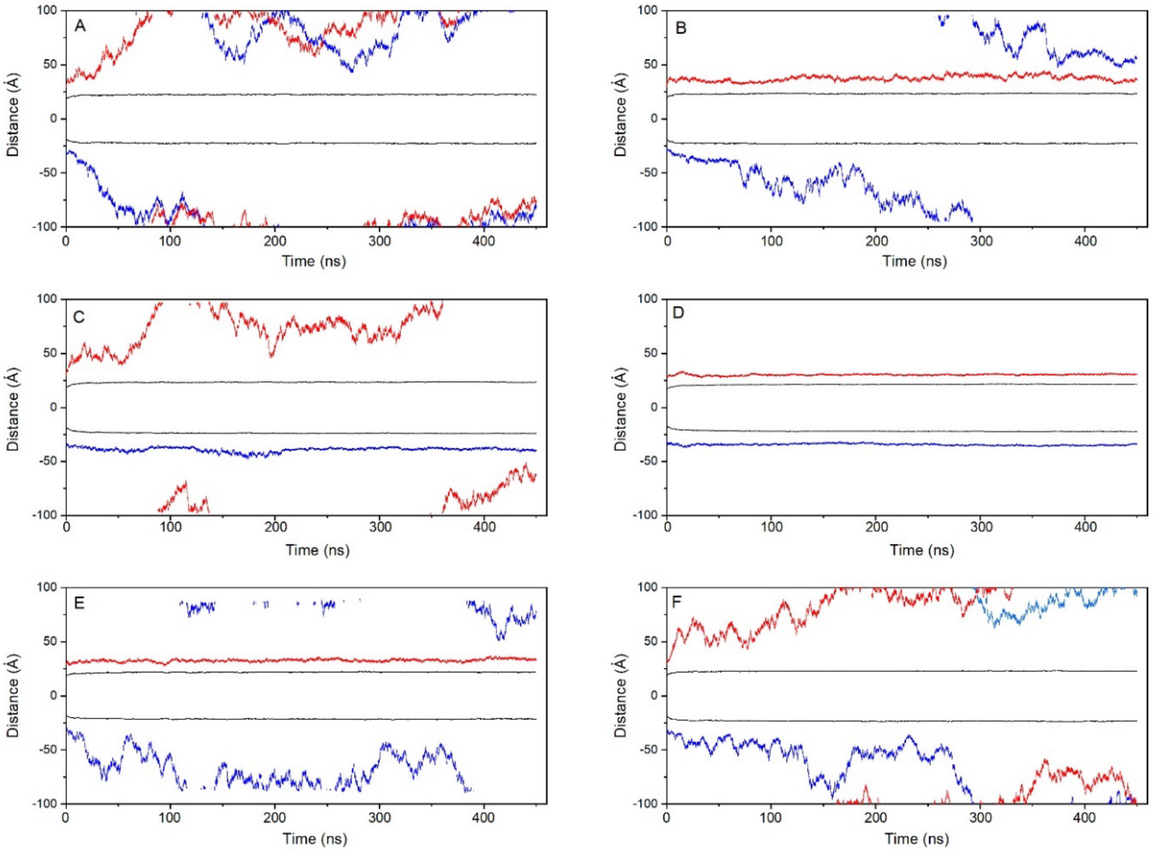

Figure S2. Time variation of the z-axis distance (center of the membrane as a reference) for systems containing (**A**) POPC/CHOL, (**B**) POPE/CHOL, (**C**) POPS/CHOL, (**D**) PI-3P/CHOL, (**E**) BMGP/CHOL and (**F**) PSM/CHOL in the presence of 0.15 M $CaCl_2$ (MD time 450 ns). The global z-axis distance of the two FP peptides in the system are depicted in blue and red line colours, whereas the z-axis distance of the phosphate atoms of the phospholipids, which define the membrane surface, is depicted in black (upper and lower boundaries).

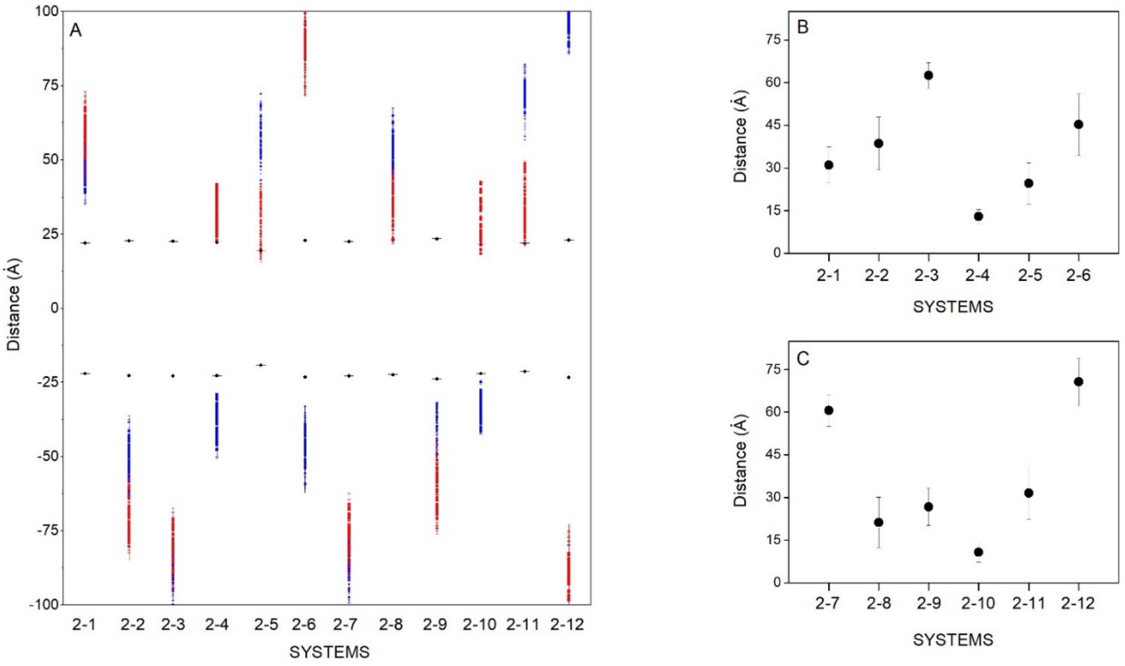

Figure S3. (**A**) Average z-axis distance for the CA carbon atoms of the two FP peptides in each one of the systems (-■- and -■- for peptides 1 and 2, respectively) in relation to the average z-axis distance for the phosphate atoms of the phospholipids (-●-). (**B, C**) Average z-axis distance separation for all the CA carbon atoms of the two FP peptides to the average z-axis distance for the phosphate atoms of the phospholipids as a reference in the presence of (**B**) 0.15 M NaCl or (**C**) 0.15 M $CaCl_2$. The systems correspond to POPC/CHOL (2-1, 2-7), POPE/CHOL (2-2, 2-8), POPS/CHOL (2-3, 2-9), PI-3P/CHOL (2-4, 2-10), BMGP/CHOL (2-5, 2-11) and PSM/CHOL (2-6, 2-12) in the presence of either (2-1 to 2-6) 0.15 M NaCl or (2-7 to 2-12) 0.15 M $CaCl_2$. The data represent the average for the last 20 ns of the MD simulation.

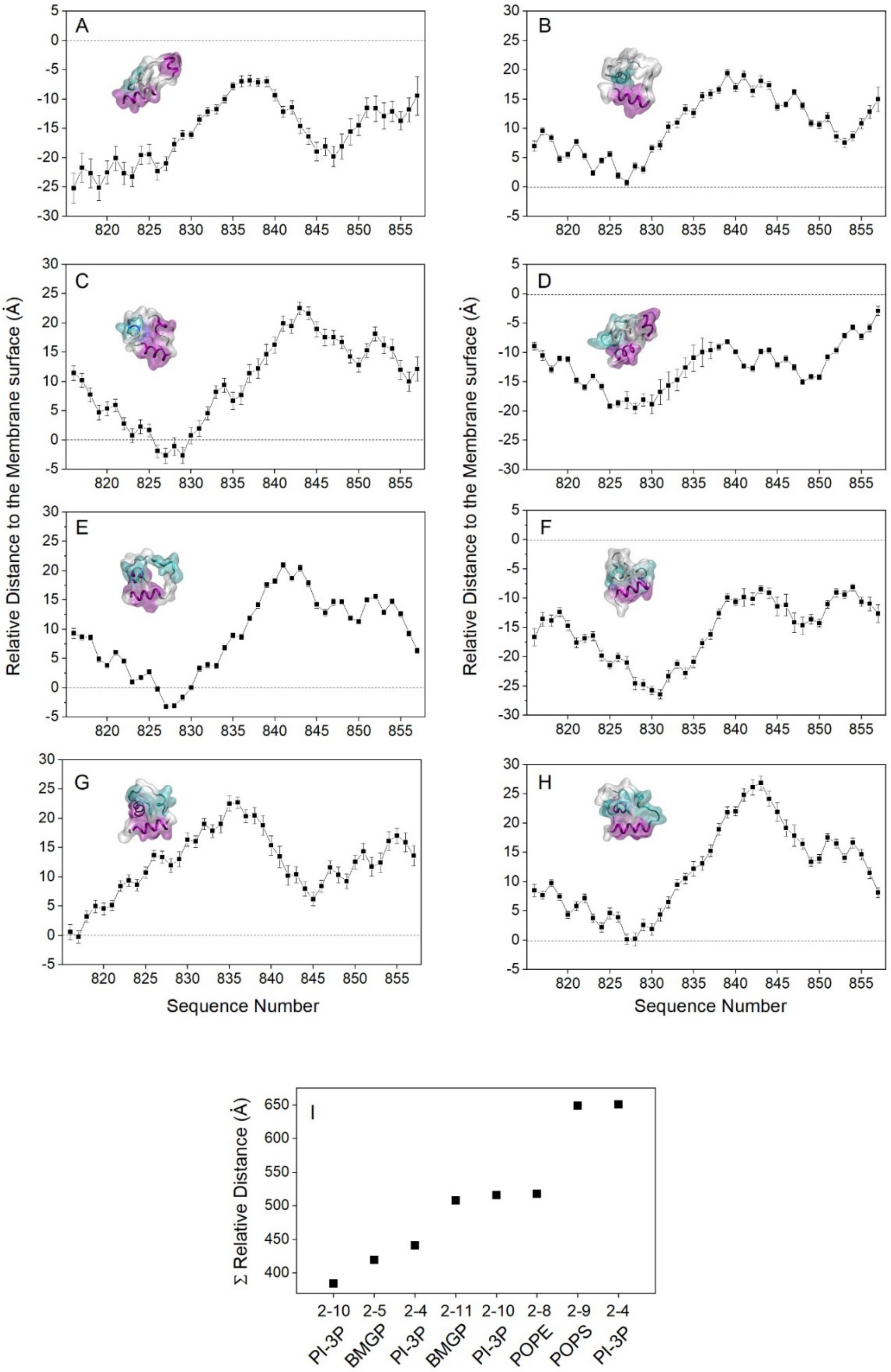

Figure S4. Average relative z-axis distance for the CA carbon atoms for each one of the residues of the FP peptides which are bound to the membrane surface along the MD simulation. Each peptide belong to systems (**A**) 2-4, (**B**) 2-4, (**C**) 2-5, (**D**) 2-10, (**E**) 2-10, (**F**) 2-9, (**G**) 2-8 and (**H**) 2-11. (**I**) Sum of the relative CA atoms z-axis distance for the specified phospholipids in each system. The data represent the average for the last 20 ns of the MD simulation.

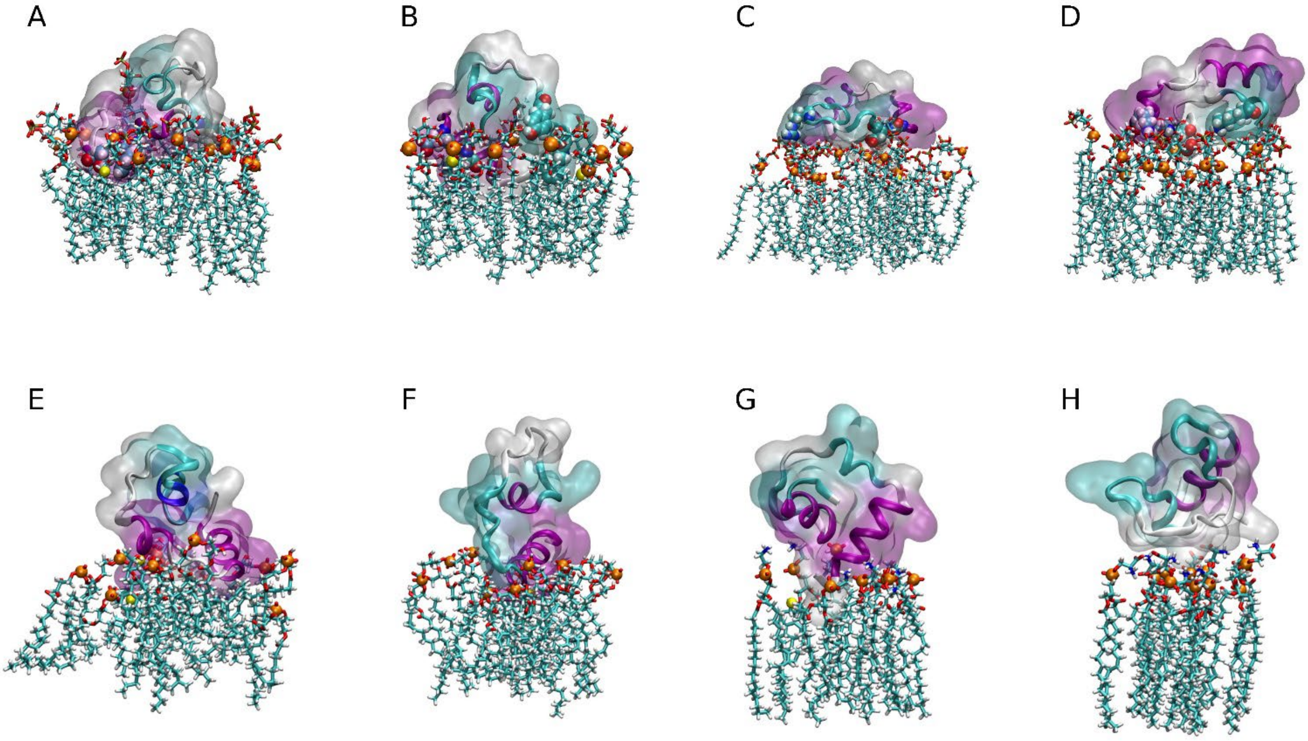

Figure S5. Final composition of the FP peptides in the systems (**A**) 2-4, (**B**) 2-10, (**C**) 2-10, (**D**) 2-4, (**E**) 2-5, (**F**) 2-11, (**G**) 2-8 and (**H**) 2-9. The peptides are shown in ribbon and transparent surface drawing style with coloured secondary structures (white (coil), violet (helix), yellow (extended), light blue (turns)). The amino acid residues forming hydrogen bonds with the PI-3P phospholipid are shown in VDW style. The lipids surrounding the peptides at a distance of 5A or less are also shown in licorize drawing style. The phospholipid phosphate and the CHOL oxygen atoms are shown in VDW drawing style (orange and yellow, respectively). The other lipids, water an ions have been removed for clarity.

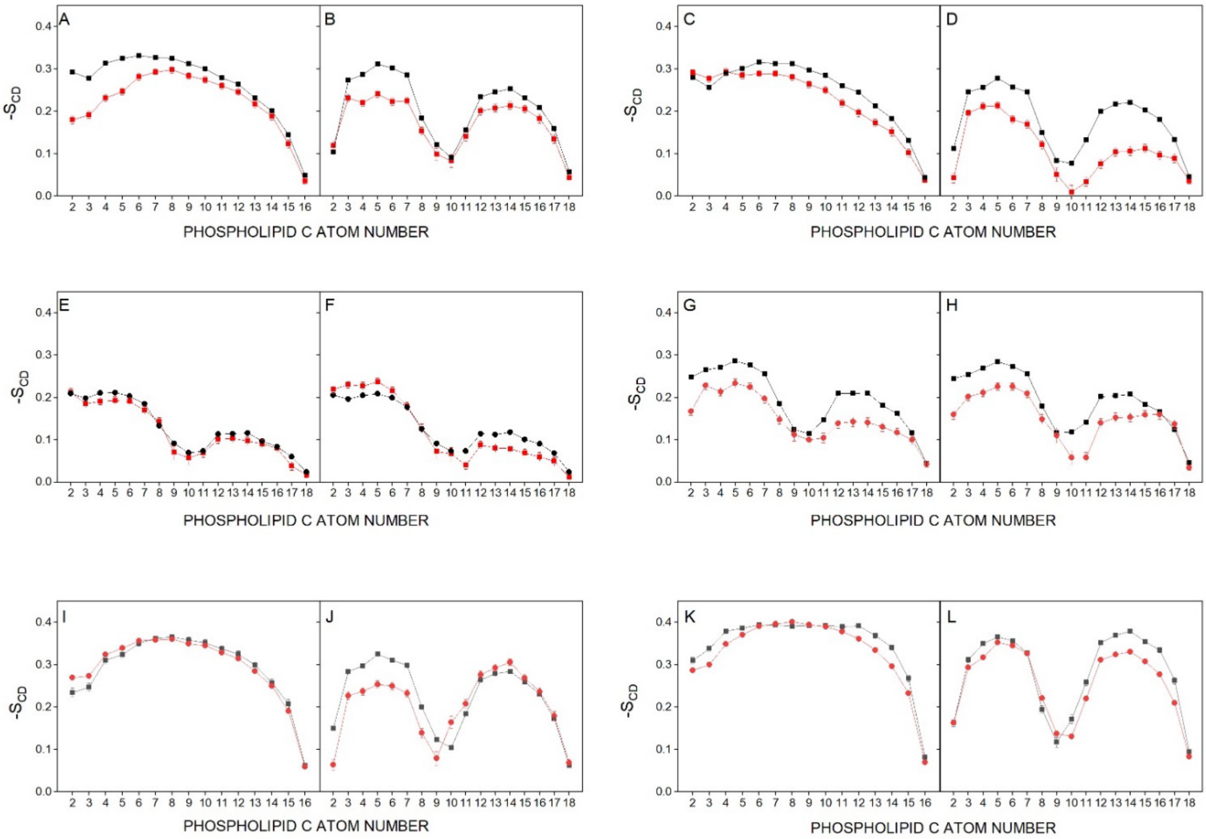

Figure S6. Average deuterium order parameter $-S_{CD}$ computed for the hydrocarbon acyl chains of the phospholipids for systems (**A,B**) 2.4, (**C,D**) 2.10, (**E,F**) 2.5, (**G,H**) 2.11, (**I,J**) 2.8, and (**K,L**) 2.9. (**A,C,I,K**) palmitoyl and (**B,D,E,F,G.H,J,L**) oleoyl acyl chains of (**A,B,C,D**) PI-3P, (**E,F,G,H**) BMGP, (**I,J**) POPE and (**K,L**) POPS. The data correspond to the bulk phospholipid acyl chains (-■-) and the phospholipid acyl chains within 5 Å of the FP (-■-). The analysis was carried out for the last 20 ns of simulation.

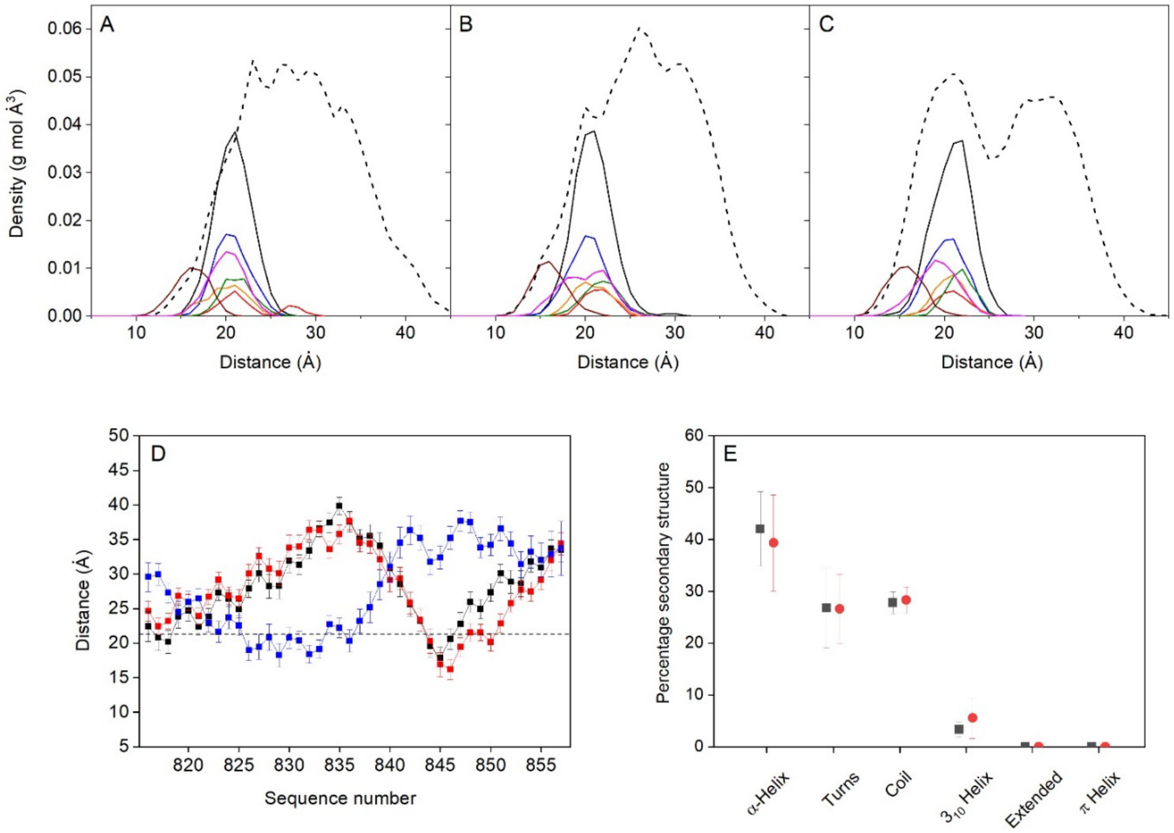

Figure S7. Mass density profiles for the last 30 ns of the MD simulation for (**A**) system 3-1, (**B**) system 3-2 and (**C**) system3-3. The mass density profiles correspond to the phosphate atoms of POPC (black), POPE (blue), POPS (red), PI-3P (orange), PSM (olive), BMGP (magenta), the oxygen atom of CHOL (wine) and the FP peptide (dotted black line). (**D**) Average relative z-axis distance for the CA carbon atoms for each one of the residues of the FP peptides (systems 3-1 (black), 3-2 (red) and 3-3 (blue) for the last 30 ns of the MD simulation (the dotted line represents the average COM of the phosphate atoms of the phospholipids). (**E**) Average percentage secondary structure for the first (■ black) and the last (● red) 30 ns of simulation.

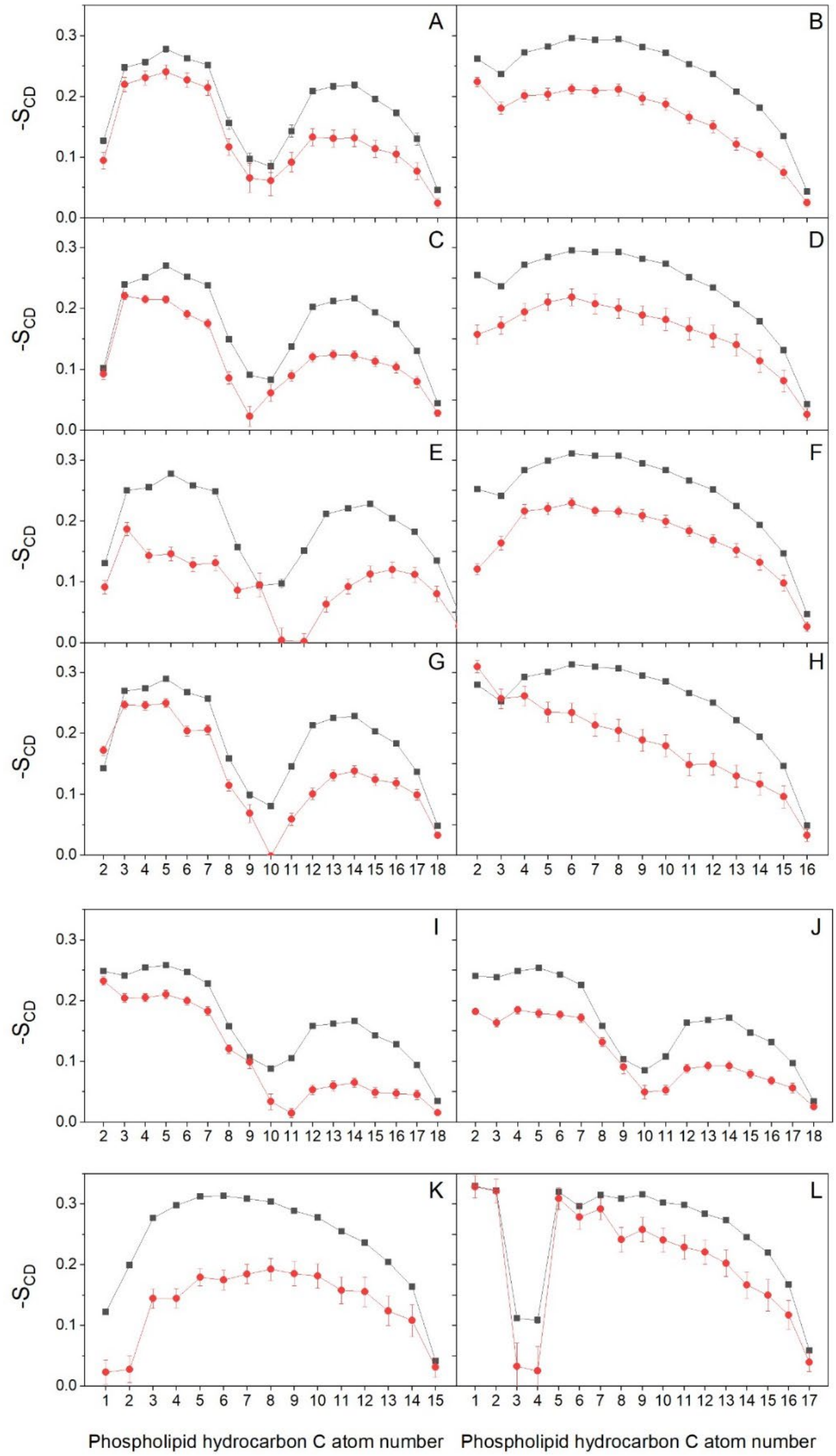

Figure S8. Average deuterium order parameter –SCD computed for the hydrocarbon acyl chains of the phospholipids for systems 3-1, 3-2 and 3-3 over the last 30 ns of simulation. (**A,C,E,G**) oleoyl and (**B,D,F,H**) palmitoyl acyl chains of (**A,B**) POPC, (**C,D**) POPE, (**E,F**) POPS, (**G,H**) PI-3P, (**I**) 3- and (**J**) 3′-oleoyl chains of BMGP and (**K**) palmitoyl and (**L**) sphyngosyl acyl chains of PSM. The data correspond to the bulk phospholipid acyl chains (-■-) and the phospholipid acyl chains within 5 Å of the FP (-●-).

Table S1. Systems and components used in the first step of this study (FP in water). The NaCl (system 1-1) and $CaCl_2$ (system 1-2) concentration was 0.15M. The MD production trajectories for each one of the systems were computed for 150 ns.

	**SYSTEM 1-1**	**SYSTEM 1-2**
**MD time**	150 ns	150 ns
**No. H_2O**	33916	33822
**No. Na^+**	100	-
**No. Ca^{2+}**	-	98
**No. Cl^-**	96	192
**No. peptides**	2	2
**x/y/z Å (final)**	99:91:142	101:91:143
**FP**<br>**+**<br>**ions**<br>**+**<br>**water**<br><br>**(t = 0 ns)**	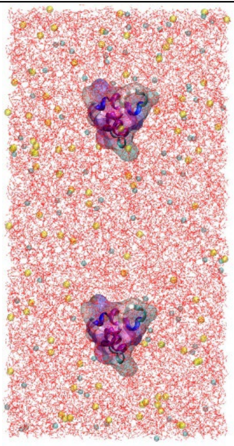	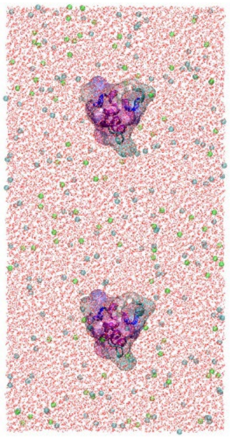

* The peptides are shown in both ribbon and surface transparent drawing style with coloured secondary structures, whereas the surrounding water molecules and ions are shown in lines and VDW drawing style, respectively.

Table S2. Systems and components used in the second step of this study (FP peptides in the presence of the membrane systems). The NaCl and $CaCl_2$ concentration was 0.15M. Membrane thickness at the end of the MS simulation is also shown. The MD production trajectories for each one of the systems were computed for 450 ns.

	SYSTEM 2-1	***SYSTEM 2-2***	***SYSTEM 2-3***	***SYSTEM 2-4***	***SYSTEM 2-5***	***SYSTEM 2-6***
**H_2O**	24644	22258	22059	23048	21691	21950
**Na^+**	74	67	217	521	65	216
**Cl^-**	70	63	63	67	61	62
**Peptides**	2	2	2	2	2	2
**Phospholipid (number)**	POPC 150	POPE 150	POPS 150	PI-3P 150	PSM 150	BMGP 150
**No. CHOL**	50	50	50	50	50	50
**Thickness Å**	44.4	45.3	46.3	44.6	46	38.9
**x:y:z Å (final)**	91:84:193	82:81:187	83:78:201	85:86:195	88:79:203	102:100:150
	SYSTEM 2-7	***SYSTEM 2-8***	***SYSTEM 2-9***	***SYSTEM 2-10***	***SYSTEM 2-11***	***SYSTEM 2-12***
**H_2O**	24576	22197	22073	23568	21632	21965
**Ca^{2+}**	72	65	140	294	63	139
**Cl^-**	140	126	126	134	122	124
**Peptides**	2	2	2	2	2	2
**Phospholipid (number)**	POPC 150	POPE 150	POPS 150	PI-3P 150	PSM 150	BMGP 150
**CHOL**	50	50	50	50	50	50
**Thickness Å**	45	45.8	47.2	43.9	46.4	43.5
**x:y:z Å (final)**	89:86:199	81:81:192	77:82:198	88:86:187	85:76:227	85:91:177

Table S3. Systems and components used in the third step of this study (FP peptides in the presence of a biological model membrane). The NaCl concentration was 0.15M. Membrane thickness at the end of the simulation is also shown. The MD production trajectories for each one of the systems were computed for 800 ns.

	SYSTEM 3-1		***SYSTEM 3-2***		***SYSTEM 3-3***		
**H_2O**	10626		10603		10597		
**Na^+**	99		99		99		
**Cl^-**	30		30		30		
**Peptides**	1		1		1		
	Monolayer		Monolayer		Monolayer		* Lipid area Å^2 (mean ± sd)
	TOP	*LOWER*	*TOP*	*LOWER*	*TOP*	*LOWER*	
**POPC**	32	36	33	36	33	36	56.6 ± 0.5
**POPE**	14	16	14	16	14	16	54.8 ± 1.3
**POPS**	4	4	4	4	4	4	55.3 ± 1.9
**PI-3P**	6	6	6	6	6	6	54.1 ± 1.2
**PSM**	6	6	6	6	6	6	51.8 ± 1.6
**BMGP**	11	12	11	12	11	12	70.4 ± 1.5
**CHOL**	16	20	17	20	17	20	27.5 ± 0.7
TOTAL	189		191		191		
**Thickness Å**	42.6		43.7		43.5		
**x:y:z Å (final)**	87:83:106		87:89:110		86:85:108		

* Average over the last 30 ns of the MD simulation.

Table S4. Number of contacts and hydrogen bonds between the FPs with the lipids for the selected membrane systems (rounded to the whole number, two FP´s per system). The data represent the average of the last 20 ns of the MD simulation.

				**CONTACTS**				
**SYSTEMS**	PI-3P	CHOL	BMGP	CHOL	POPE	CHOL	POPS	CHOL
2-4	4498 ± 236	3 ± 3	-	-	-	-	-	-
2-4	600 ± 197	0	-	-	-	-	-	-
2-5	-	-	2564 ± 234	89 ± 47	-	-	-	-
2-8	-	-	-	-	1088 ± 152	0		
2-9	-	-	-	-	-	-	293 ± 56	0
2-10	3201 ± 239	442 ± 91	-	-	-	-	-	-
2-10	1436 ± 64	0	-	-	-	-	-	-
2-11	-	-	1027 ± 252	206 ± 79	-	-	-	-
				**HYDROGEN BONDS**				
**SYSTEMS**	PI-3P	CHOL	BMGP	CHOL	POPE	CHOL	POPS	CHOL
2-4	9 ± 1	0	-	-	-	-	-	-
2-4	14 ± 4	0	-	-	-	-	-	-
2-5	-	-	0	0	-	-	-	-
2-8	-	-	-	-	0	0	-	-
2-9	-	-	-	-	-	-	0	0
2-10	10 ± 1	0	-	-	-	-	-	-
2-10	16 ± 1	0	-	-	-	-	-	-
2-11	-	-	0	0	-	-	-	-

Table S5. Number of lipids around the FP up to 5 Å and contacts between the FPs and lipids. The data represent the average for the three systems and for the last 30 ns of the MD simulation.

	LIPIDS AROUND FP	CONTACTS
**POPC**	7.9 ± 2.1	989.4 ± 378.8
**POPE**	4.3 ± 1.1	324.5 ± 206.6
**POPS**	0.8 ± 0.5	390.3 ± 212.7
**PI-3P**	0.8 ± 0.7	17.1 ± 14.2
**PSM**	1.2 ± 0.3	334.5 ± 133.4
**BMGP**	3.0 ± 1.1	695.2 ± 281.5
**CHOL**	2.7 ± 1.2	197.5 ± 104.1
